# Audiovisual integration in the McGurk effect is impervious to music training

**DOI:** 10.1038/s41598-024-53593-0

**Published:** 2024-02-08

**Authors:** Hsing-Hao Lee, Karleigh Groves, Pablo Ripollés, Marisa Carrasco

**Affiliations:** 1 Department of Psychology, New York University, New York, USA; 2 Center for Language, Music, and Emotion (CLaME), New York University, New York, USA; 3 Music and Audio Research Lab (MARL), New York University, New York, USA; 4 Center for Neural Science, New York University, New York, USA

**Keywords:** Human behaviour, Sensory processing

## Abstract

The McGurk effect refers to an audiovisual speech illusion where the discrepant auditory and visual syllables produce a fused percept between the visual and auditory component. However, little is known about how individual differences contribute to the McGurk effect. Here, we examined whether music training experience—which involves audiovisual integration—can modulate the McGurk effect. Seventy-three participants completed the Goldsmiths Musical Sophistication Index (Gold-MSI) questionnaire to evaluate their music expertise on a continuous scale. Gold-MSI considers participants’ daily-life exposure to music learning experiences (formal and informal), instead of merely classifying people into different groups according to how many years they have been trained in music. Participants were instructed to report, via a 3-alternative forced choice task, “what a person said”: /Ba/, /Ga/ or /Da/. The experiment consisted of 96 audiovisual congruent trials and 96 audiovisual incongruent (McGurk) trials. We observed no significant correlations between the susceptibility of the McGurk effect and the different subscales of the Gold-MSI (active engagement, perceptual abilities, music training, singing abilities, emotion) or the general musical sophistication composite score. Together, these findings suggest that music training experience does not modulate audiovisual integration in speech as reflected by the McGurk effect.

## Introduction

Speech perception involves both auditory (e.g., the sound waves) and visual signals (e.g., the mouth of the speaker). Thus, audiovisual integration shapes our perception of speech and further influences how we communicate with each other in our daily lives. The McGurk effect shows how visual input can reshape auditory perception by pairing an auditory syllable with an incongruent visual syllable. Instead of the original auditory stimulus, a fused, not presented syllable, will be perceived. For example, if a visual /Ga/ input is combined with the auditory /Ba/ sound, a /Da/ sound is often perceived by the observer^1^.

The McGurk effect can be a useful tool to evaluate the audiovisual integration ability in speech perception^2,3^. For example, researchers have used the susceptibility of the McGurk effect, i.e., how prone people are to report the fused percept, to evaluate audiovisual integration in different populations, such as people with amblyopia^4^, autism spectrum disorder^5^, and in cochlear-implanted deaf subjects^6^.

Despite the prevalence of the McGurk effect, there are pronounced individual differences that modulate the strength of the effect^7–9^. For example, the frequency with which an individual reports a McGurk illusion varies as a function of the people pronouncing the stimuli (e.g., gender and voice^7^). Additionally, whether individuals perceive an illusion depends on their disparity threshold, which refers to the point at which the noise in one modality is high enough to prevent the fused percepts. Thus, when the perceived disparity falls below this threshold, an observer will assume these two modalities come from the same source and integrate them, which leads to a McGurk effect. In contrast, when the perceived disparity is above this threshold, the original auditory syllable will be perceived^7^. In addition to the experimental setup^7,8^, genetic and environmental factors^10^, and neural measures (e.g., brain activity in the left superior temporal sulcus^11^), can lead to the individual differences in the susceptibility to the McGurk effect.

Cognitive and perceptual abilities have been evaluated with regard to the susceptibility of the McGurk effect^12^. None of the measurements (i.e., perceptual gradiency, attentional control, processing speed, and working memory capacity) could predict the susceptibility of the McGurk effect, and only a small amount of the variability could be explained by individual differences in lipreading skills. What other factors could underlie the individual differences observed in the McGurk effect?

A critical factor that influences audiovisual integration in general is music training. It has been reported that musically trained individuals are faster and more accurate in processing concurrent audiovisual information than non-musicians in a double-flash illusion task, suggesting intensive music training can alter multisensory integration^13^. Likewise, musicians have a narrower temporal integration window for audiovisual processing than non-musicians, leading to a more accurate response to an audiovisual simultaneity judgment task^14^. Neuroimaging findings also suggest that music training can alter the structural^15^ and functional^16^ brain networks supporting audiovisual processing.

However, it is unclear whether the benefits of musicians in audiovisual integration generalize to speech perception. On the one hand, the benefits may only exist in pure tone or music perception but not in speech perception. For instance, piano practicing participants have a narrower temporal integration window for music, but not for speech perception^17^. At the neural level, musicians showed increased audiovisual asynchrony responses for music more than for speech in several superior-temporal-sulcus-premotor-cerebellar circuitry, which is critical for multisensory integration. This study suggests that musical training modulates the audiovisual temporal relation only for music and not for speech^17^. On the other hand, Jain et al.^18^ have shown that short-term music training can improve performance in a speech perception task, and this finding generalizes to older populations^19^ and people with cochlear implants^20^. Recent research also shows that musical training modulates auditory-motor coupling in the context of synchronization between speech perception and production^21,22^.

Thus, there are two possible hypotheses regarding how music learning experience may shape the McGurk effect: First, if music learning shapes the audiovisual integration of speech perception, this would lead to a lower susceptibility of the McGurk effect, as people with more music expertise may rely more on the auditory features. Second, if the influence of music learning on audiovisual integration only manifests in music but does not transfer to speech perception, the susceptibility of the McGurk effect would be similar across individuals with different degrees of musical expertise.

The two previous studies that have examined whether musicians and non-musicians have different susceptibility to the McGurk effect hold contradictory conclusions (see Table 1). The first study examined the susceptibility of the McGurk effect in Italian musicians and non-musicians^23^. They compared the accuracy in the McGurk task and found that musicians and non-musicians had comparable accuracy in the auditory-only condition, while the musicians had a higher accuracy in the audiovisual incongruent condition (i.e., the McGurk condition), indicating that musicians could identify the auditory input more accurately than the non-musicians and be less susceptible to the McGurk illusion. They concluded that musicians are less susceptible to the McGurk illusion due to their training in auditory perception. They hypothesized that musicians assign a higher weight to the auditory input when the auditory and visual inputs mismatch. However, the comparison may be limited because they adopted a between-subject design to compare the accuracy across conditions (i.e., the musicians in the auditory-only and audiovisual condition were different groups of participants). It is possible that the null-difference in the auditory-only condition and the difference in the audiovisual condition stems from between-subject differences rather than the music training experience. Additionally, they also had a relatively small sample size: 20 musicians and 20 non-musicians in each condition. Their findings, notwithstanding some caveats, are worth further investigation to clarify whether musicians are indeed less likely to be susceptible to the McGurk effect.

**Table 1:** The comparisons between current study and the previous studies.

	Proverbio, Massetti, Rizzi & Zani (2016)	Politzer-Ahles & Pan (2019)	Current study
Conclusion	No McGurk effect in musicians	Stronger McGurk effect in musicians than non-musicians	Music training does not affect the McGurk effect
Stimuli	Audiovisual + auditory only	audiovisual congruent + audiovisual incongruent + auditory only	audiovisual (congruent + incongruent)
Task instruction	Write down “what they had heard”	answer through keyboard “what sound they believed they heard”	Answer what the person said (forced choice)
Groups in different conditions	Between-subject	within-subject	Within-subject
Musician definition	Average 23.4 years of music learning experience	at least 13 years of training	A continuous scale (Gold-Musical Sophistication Index)
Non-musicians inclusion	Lack of musical studies and specific interest in music as a hobby	No music training within the past 10 years	A continuous scale (Gold-Musical Sophistication Index)
Country/Native Language	Italy/Italian	Hong Kong/Mandarin or Cantonese	USA/Multiple (e.g., Arabic, English, French, Hindi, Italian, Japanese, Korean, Mandarin, Spanish, Turkish)
Sample size (Musicians/Non-musicians)	20/20	62/62	73 (continuous scale) 30/43 based on the United States norm in music training
Gender (Male/Female)	16/24	22/102	26/45/1 (non-binary) /1 (not reported)
Stimuli background noise	No background noise	With background noise	no background noise
Dependent variable	Accuracy of the task	Accuracy of the task	Accuracy of the task and the proportion of the McGurk effect
Number of speakers	2	1	8
Number of syllables tested	8	8	3 basic McGurk syllables
Proportion of the congruency	64 congruent auditory, 64 incongruent (per group)	8 congruent, 56 incongruent	96 congruent, 96 incongruent
Presentation approach	Powerpoint	DMDX	MATLAB
Analysis	ANOVA on arcsine-transformed data	Generalized linear mixed-effects models	Regression
Inter-trial interval	5 s	4 s	Self-paced
Distance from the monitor	80 cm	Not controlled	57 cm
Video size	Not reported	Not reported	12.5° × 10°
Eye-tracking	Not reported	Not reported	Recorded for 2/3 of the participants
Response coding	Manual	Automated (based on the first character of response)	Automatic
McGurk trial number	8 stimuli × 8 times	8 stimuli × 8 times	8 stimuli × 12 times
Experiment administration	Not reported	Group	Individual

The second study examining musical training revealed the opposite findings^24^. The authors assessed the McGurk effect using a different stimulus set with musicians and non-musicians whose language was Mandarin or Cantonese. Interestingly, they found that musicians were more susceptible to the McGurk effect. To be noted, they used a within-subject design across different testing conditions and had (unplanned) higher background noise than Proverbio et al.^23^ in their auditory and audiovisual stimuli.

The opposite findings in these two studies involve populations from different countries with speakers of different languages, among other differences in the experimental procedure and stimuli used. In Table 1 we compare the different experimental designs. We note that it is unlikely that the different language is responsible for the differential effects, as it has been shown that speakers of different languages (e.g., Chinese and English) are similarly susceptible to the McGurk effect^25^.

In any case, it is unclear whether and how music training reshapes susceptibility (if any) to the McGurk effect. Importantly, these previous studies compared musicians versus non-musicians, without considering that musical training is a continuum. This inconsistency motivated us to revisit this question and evaluate if there is any difference of audiovisual integration in speech as reflected by the McGurk effect throughout the music learning trajectory.

We used a more diverse group of participants and assessed the music learning experience using a continuous measure quantifying both formal and informal musical training experience: the Goldsmiths Musical Sophistication Index (Gold-MSI)^26^, instead of binarily classifying participants into musicians or non-musicians. Gold-MSI is an inventory that considers a wide range of facets of the music learning experience; it measures musical sophistication which is conceptualized as the musical ability, skills, achievements, and music-related behaviors (including, for example, the ability to communicate about music at a higher level), to use music, to induce or manipulate emotional states, and to compare music styles. Thus, unlike other traditional assessments for musicality that only consider objective aspects, the Gold-MSI considers both subjective and objective aspects of music learning experience. In addition, the Gold-MSI self-report considers the music skills developed through non-explicit or non-formal means, including repeated and focused engagement (such as self-practice) with music.

Gold-MSI has five different subscales, including *active engagement*, *perceptual abilities*, *musical training*, *singing abilities*, and *emotions*. This self-report includes a *general musical sophistication* score that is computed from selective items of the questionnaire. Previous studies have shown that Gold-MSI can predict the synchronization of speech perception and speech production, with high synchronizers showing higher scores in all the Gold-MSI subscales (except the emotion one) than low synchronizers^21,22^.

The Gold-MSI was used in the current study because it serves as a good predictor regarding whether and how music learning modulates human behaviors. Indeed, a previous study encouraged researchers to use such a standardized procedure to evaluate people’s musicality so that future studies can have a standard to repeat and conduct meta-analyses^27^. The music training subscale of the Gold-MSI has been linked to underlying neural circuits. For example, the subscales are positively correlated with the volume of posterior cingulate cortex, insula, and medial orbitofrontal cortex in older adults, which are brain regions involved in cognitive control, memory, language, and emotion^28^. Additionally, the Gold-MSI informs how music training benefits older populations by enhancing the connectivity of these circuits^29^.

In sum, here we examined whether music learning experience, as indexed by the Gold-MSI, can predict participants’ susceptibility to the McGurk effect.

## Methods

### Participants

Seventy-three people (age range 18–66, mean: 25.97, SD = 7.19, median = 25; 26 males, 45 females,1 non-binary, 1 preferred not to report) participated in this experiment. We determined our sample size based on studies that also examined individual differences in the McGurk effect (i.e.,^8,30^). A sample size of 30 participants is considered sufficient for correlation analysis^31^. Additionally, according to MorePower^32^, with 73 participants, we have 80% power to detect correlations as low as 0.32. All participants were naive to the purpose of the experiment and provided informed consent before the experiment. All participants were free from neurological disorders and hearing issues and had normal or corrected-to-normal vision. The experimental procedures were in line with the Helsinki declaration and approved by the University Committee on Activities involving Human Subjects at New York University. Participants received course credit or were paid for their participation.

### Stimuli

The experimental stimuli were the same as those used in Basu Mallick et al.^7^ and were downloaded from https://openwetware.org/wiki/Beauchamp:Stimuli. The stimuli were centered and extended 12.5° × 10° on the screen and were presented using MATLAB (MathWorks, Natick, MA). The stimuli contained 8 speakers producing both audiovisual congruent and incongruent trials. For the congruent trials, both the visual (face including mouth) and auditory stimuli produce /Ga/, /Ba/, and /Da/ syllables. In the incongruent trials, the visual input was /Ga/ while the auditory input was /Ba/.

Eye movement data was recorded from 70% of participants using EyeLink 1000 (SR Research, Osgoode, Ontario, Canada) at a sampling rate of 1000 Hz, to ensure that they were looking at the stimuli throughout the trial. If observers moved their gaze beyond the stimulus location the trial would be aborted and repeated at the end of the block. Data for the other 30% of participants were collected using the same experimental setup, but without an eye-tracker device; however, the experimenter ensured that participants were looking at the screen.

### Procedure

Participants were instructed to place their chin on the chinrest, which was 57 cm away from the monitor. Participants completed 3 practice trials to ensure that they understood the task instructions. The practice trials were audiovisual congruent trials where the same speaker said /Ba/, /Ga/ and /Da/. Participants were provided 3 options (1) /ba/, (2) /ga/, and (3) /da or tha/, via response keys 1, 2, and 3, respectively. The response keys were the same throughout the experiment, and participants were instructed to use their left ring finger, middle finger, and index finger to respond. They were told to respond to what the person said and to look at the screen all the time. If they were not sure about the answer, they were instructed to make their best guess, without a time limit to respond. The instruction was designed to avoid emphasizing either modality so that participants would not be biased toward any particular response^2^. Note that the speaker in the practice trials was not included in the experiment. After the practice, participants were asked if they could hear the sound and see the speaker’s entire face clearly and if they needed to adjust the volume before they started the experiment.

In the experiment, there were two blocks of 96 trials each. Among the 192 trials, there were 96 McGurk trials (i.e., marked as /MG/, the audiovisual incongruent trials), in which the speaker’s lip pronounced /Ga/ and the auditory sound was /Ba/. The other 96 trials were all audiovisual congruent trials, 1/3 /Ba/, 1/3 /Ga/, and 1/3 /Da/. The procedure was the same as in the practice trials. Different types of stimuli were interleaved in a random order. The experiment lasted around 15–20 min.

### Gold-MSI questionnaire

The Goldsmiths Musical Sophistication Index (Gold-MSI) is a questionnaire that comprises both subjective self-assessment and objective measurements to music training experience^26^. Gold-MSI contains five different subscales including active engagement (9 items), perceptual abilities (9 items), musical training (7 items), singing abilities (7 items), and emotions (6 items). Additionally, a general musical sophistication (GS) score can be computed from selective items from the five subscales (18 items). Each item score ranges from 1 to 7. The norms (mean) for people in the United States (n = 147,633) in each subscale are: active engagement (42), perceptual abilities (50), music training (27), singing skills (32), emotions (35) and general sophistication (82)^26^. The means from our samples were: active engagement (40), perceptual abilities (45), music training (25), singing skills (28), emotions (32) and general sophistication (75). Although the obtained scores seem slightly lower than the norm, they are similar to scores reported in other studies (e.g.^22,33,34^, see Table S1).

## Results

**Figure 1:**
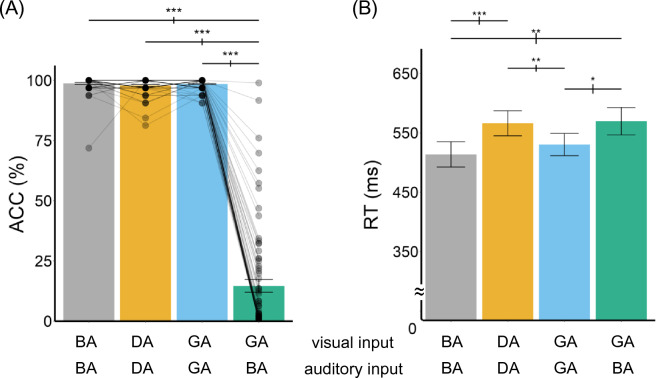
(**A**) Response accuracy and (**B**) reaction time (RT) for both correct and incorrect trials in the four stimulus types. (We could not analyze only the correct responses because for ~ 1/3 of the participants, there were no correct responses for the McGurk trials). The first row of the x-axis indicates the visual input, and the second row indicates the auditory input. Each dot indicates a participant’s response. The error bars indicate one s.e.m. ***p < 0.001, **p < 0.01, *p < 0.05.

We first performed a one-way analysis of variance (ANOVA) on the accuracy (i.e., whether the participant correctly identified the auditory syllable) for different types of stimuli (congruent and incongruent) to verify participants had no difficulty in answering the percept of audiovisual congruent trials (i.e., /BA/, /GA/, /DA/; Fig. 1A). A significant main effect of stimulus type was observed [*F*(3, 216) = 927, *p* < 0.001, *η*^*2*^ = 0.93]. Post-hoc analyses revealed that all the congruent trials showed higher accuracy than the McGurk trials[/Ba/ and /MG/: [*t*(72) = 30.66, *p* < 0.001, *d* = 3.59; /Da/ and /MG/: *t*(72) = 30.51, *p* < 0.001, *d* = 3.57; /Ga/ and /MG/: *t*(72) = 31.88, *p* < 0.001, *d* = 3.73], but there were no differences among the congruent stimuli: /Ba/ and /Da/ [*t*(72) = 1.45, *p* = 0.15, *d* = 0.17], /Da/ and /Ga/ [*t*(72) = 1.32, *p* = 0.192, *d* = 0.15] or /Ba/ and /Ga/ [*t*(72) = 0.51, *p* = 0.61, *d* = 0.06]. This analysis shows that participants had similar performance and had no problems in identifying the auditory signals in the audiovisual congruent trials but had a significant illusion in the McGurk (audiovisual incongruent) trials.

The same pattern of results was obtained when we arc-sine transformed the data to avoid ceiling effects^23^. A significant main effect of stimulus type was observed [*F*(3, 216) = 755.8, *p* < 0.001, *η*^*2*^ = 0.91]. Post-hoc analyses revealed that all the congruent trials showed higher accuracy than the McGurk trials[/Ba/ and /MG/: [*t*(72) = 29.45, *p* < 0.001, *d* = 3.45; /Da/ and /MG/: *t*(72) = 28.56, *p* < 0.001, *d* = 3.34; /Ga/ and /MG/: *t*(72) = 31.99, *p* < 0.001, *d* = 3.74], but there were no differences between /Da/ and /Ga/ [*t*(72) = 1.0, *p* = 0.321, *d* = 0.12] or /Ba/ and /Ga/ [*t*(72) = 1.19, *p* = 0.239, *d* = 0.14]. A slightly higher arc-sine transformed accuracy was found in /Ba/ than /Da/ [*t*(72) = 2.1, *p* = 0.04, *d* = 0.25].

**Figure 2:**
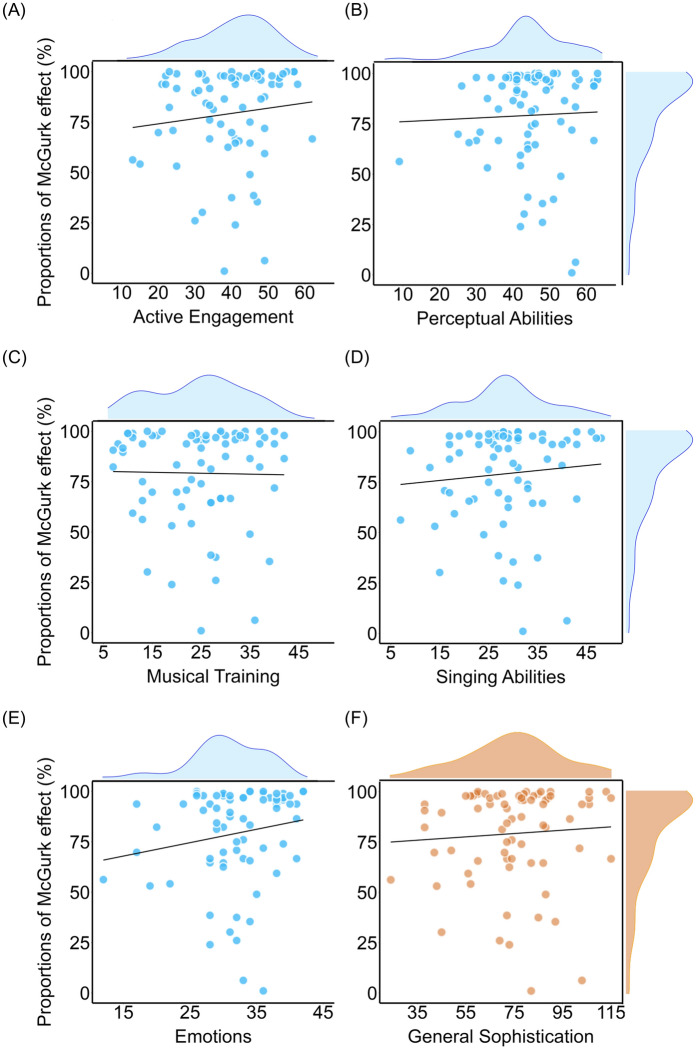
Scatterplots of the different Gold-MSI subscales with the proportion of the McGurk effect. The black lines indicate the linear fitted lines in the regression. (**A**) active engagement; (**B**) perceptual abilities; (**C**) music training; (**D**) singing abilities; (**E**) emotions; and (**F**) general musical sophistication. No significant correlations were observed between the McGurk effect and any of the six scales.

Next, we quantified the proportion of the McGurk effect and correlated it with the responses to the questionnaire. The susceptibility to the McGurk effect was calculated as the proportion for giving /Da/ as the (fused) response in the McGurk trials. The proportion of the McGurk effect ranged from 1.04 to 100% (mean = 79.21%, SD = 24.15%). The susceptibility of the McGurk effect we observed here falls right between other studies using the same materials and protocol; one had a slightly lower proportion (69% in Basu Mallick et al.^7^) and another had a slightly higher proportion (86% in Moris Fernández et al.^35^). Given the variability in the proportion of the McGurk effect in our sample, we had enough variance to examine whether music training modulated the McGurk effect. We conducted Kendall rank correlations between the proportion of the McGurk effect with the five subscales of the Gold-MSI (i.e., active engagement, perceptual abilities, musical training, singing abilities, and emotions) and with the general music sophistication score (Fig. 2).

We also provided Bayes factors calculated from JASP^36^ using default priors to verify our findings. When BF_10_ is greater than 3.00, it suggests that the observed data are at least three times as likely under the alternative than the null hypothesis. When BF_10_ is smaller than 0.33, it provides evidence that the observed data is at least 3 times more likely under the null hypothesis. When BF_10_ is between 1 to 3.00, it suggests that the data provides evidence for the alternative hypothesis that is weak or anecdotal^37^. In our analysis, no significant correlations were observed for any scale: active engagement (*τ* = 0.13, *p* = 0.10, BF_10_ = 0.62); perceptual abilities (*τ* = 0.07, *p* = 0.403, BF_10_ = 0.22); musical training (*τ* = 0.06, *p* = 0.501, BF_10_ = 0.18); singing abilities (*τ* = 0.09, *p* = 0.287, BF_10_ = 0.28); emotions (*τ* = 0.13, *p* = 0.107, BF_10_ = 0.61); general sophistication (*τ* = 0.09, *p* = 0.27, BF_10_ = 0.27). The same statistical pattern of results was observed if we only analyzed data from participants’ whose eye movements were tracked (n = 51).

As in previous studies^23,24^, we correlated the questionnaire scores with the accuracy in the McGurk trials (rather than the susceptibility of the McGurk effect). Note that it is possible for participants to make an incorrect response that was neither the correct auditory input (i.e., /BA/) nor the fused response (i.e., /DA/) by choosing the /GA/ response. There were no significant correlations between the task accuracy in the McGurk trials and the scales: active engagement (*τ* = − 0.12, *p* = 0.147, BF_10_ = 0.486); perceptual abilities (*τ* = − 0.14, *p* = 0.095, BF_10_ = 0.72); musical training (*τ* = 0.01, *p* = 0.92, BF_10_ = 0.15); singing abilities (*τ* = − 0.09, *p* = 0.27, BF_10_ = 0.3); emotions (*τ* = − 0.15, *p* = 0.075, BF_10_ = 0.9); general sophistication (*τ* = − 0.06, *p* = 0.458, BF_10_ = 0.21).

All correlations were still not significant after we considered the baseline correction to the McGurk effect proportion. Namely, the corrected illusion rate is the McGurk effect proportion minus the proportion that a participant gave /Da/ as a response to the /Ba/ congruent trials^38,39^: active engagement: *τ* = 0.11, *p* = 0.162, BF_10_ = 0.43; perceptual abilities: *τ* = 0.07, *p* = 0.422, BF_10_ = 0.22; musical training: *τ* = 0.04, *p* = 0.60, BF_10_ = 0.17; singing abilities: *τ* = 0.07, *p* = 0.374, BF_10_ = 0.23; emotions: *τ* = 0.12, *p* = 0.141, BF_10_ = 0.49; general sophistication: *τ* = 0.07, *p* = 0.406, BF_10_ = 0.21.

We also separated people into two groups based on the norm of the music training subscale in the Gold-MSI. People who scored greater than 27 (out of 49) were classified as music-experienced (n = 30); people who scored lower or equal to 27 were classified as music-inexperienced (n = 43). We then compared the proportion of the McGurk effect in these two groups using a two-sample *t*-test. Consistent with the correlation results, no significant difference between the two groups was observed [*t*(71) = 0.48, *p* = 0.635, *d* = 0.11]. Additionally, among 71 out of 73 participants who reported their gender, there was a marginally higher susceptibility in the 45 females (83.26%) than the 26 males (72.4%), [*t*(69) = − 1.84, *p* = 0.071, *d* = 0.43].

We also analyzed the reaction time (RT; Fig. 1B) data for all trials (correct and incorrect) across conditions to assess whether different types of stimuli led to a longer RT. Even though we did not instruct participants to answer as fast as possible, we conducted this exploratory analysis to assess if there were differences in the time of audiovisual integration under no time constraint. A main effect of stimulus type was observed [*F*(3, 216) = 7.01, *p* < 0.001, *η*^*2*^ = 0.09]. The post-hoc analysis revealed significant faster RT in the /Ba/ compared to /Da/ [*t*(72) = − 3.94, *p* < 0.001, *d* = 0.46] and /MG/ trials [*t*(72) = − 3.09, *p* = 0.002, *d* = 0.36], a faster /Ga/ compared to /Da/ [*t*(72) = 3.21, *p* = 0.002, *d* = 0.38] and /MG/ [*t*(72) = − 2.46,* p* = 0.016, *d* = 0.29] trials. No other differences were found (all *ps* > 0.1). Despite the slower RT in the McGurk trials, no significant correlation (all *ps* > 0.1) was observed between the Gold-MSI scales and the McGurk RTs (when participants responded /Da/ to the McGurk trials).

## Discussion

We examined whether music learning experience, as measured by the Gold-MSI, modulated participants’ susceptibility to the McGurk effect. A robust McGurk effect was observed among the participants, as they gave more incorrect responses to the auditory input in the McGurk trials than in the audiovisual congruent trials (Fig. 1A). However, neither any of the five subscales (i.e., active engagement, perceptual abilities, music training, singing abilities, and emotions) nor the general musical sophistication index of the Gold-MSI was associated with the susceptibility to the McGurk effect (Fig. 2). This finding was verified by a baseline correction, a Bayes Factor analysis, and by separating participants into two groups according to the Gold-MSI norm. We further conducted exploratory analysis on the RT data, and yet no correlation between the RT and scale ratings was observed as well.

We measured participants’ music learning experience using the Gold-MSI questionnaire, rather than merely assessing how many years participants had devoted to formal music training^23,24^. The benefits of using Gold-MSI are two-fold. First, the Gold-MSI allows for the evaluation of how other factors in addition to formal music training may predict the susceptibility of the McGurk effect. These include, for example, active engagement in music (such as attending concerts) and informal training in music (band playing). These factors were usually ignored in previous musical studies when investigating multisensory processing, yet they actually cultivate and alter our real-world listening behaviors^26^. Second, instead of comparing musicians and non-musicians in a binary manner, the Gold-MSI allows for testing a continuous measure of musical experience and sophistication. Had there been any difference between musicians and non-musicians in their susceptibility to the McGurk effect, we could have identified the critical time period or different factors.

No effect of music training on the susceptibility of the McGurk effect was found, inconsistent with the studies that reported differences, yet in different directions: The first study reported a lower susceptibility of the McGurk effect in the musicians than non-musicians^23^, whereas the second reported musicians had a higher susceptibility than non-musicians^24^. Our findings also held when we classified participants into two groups according to the norm of the music training score in the Gold-MSI. Table 1 summarizes the differences among those two studies and the current one.

To be noted, instead of merely taking response accuracy as the dependent variable as the two previous studies did, we took the proportion of McGurk response in the McGurk trials as the dependent variable, given that it is likely that participants gave an error response in the McGurk trials (e.g., /Ga/) but not the fused response (i.e., /Da/) in the McGurk trials. For example, in the current study, the susceptibility of the McGurk effect was 79.2% whereas the mean error rate of the McGurk trials was 85.34%, suggesting that participants also experienced other percept than the fused response. In addition, the two previous studies assessing music training and the McGurk effect included morphemes that are specific to their native language – Italian^23^ or Mandarin^24^. Here, we only included the basic syllables (i.e., /Ba/, /Ga/ and /Da/) included in the original McGurk task^1^. Therefore, by using these basic syllables, we were able to recruit people from a more diverse language background (e.g., Arabic, English, French, Hindi, Italian, Japanese, Korean, Mandarin, Spanish, Turkish) and make a more generalizable inference to the worldwide population regarding how music may or may not shape the audiovisual integration in speech as reflected by the McGurk effect.

The different findings in the current study compared to the previous two studies are unlikely to be due to the different spoken languages. Although there is no consensus whether the McGurk effect would be influenced by the spoken language^2^, a large sample study has shown that English and Mandarin speakers do not differ in their susceptibility to the McGurk effect^25^. Additionally, some previous studies reported inconsistent findings primarily due to the low sample size and the low statistical power they had^25^.

Finally, our experimental stimuli were well-controlled and have been widely used in previous studies (e.g.,^7,35,40^). We included all possible combinations of the syllables (i.e., /Ba/, /Ga/, /Da/) in the audiovisual congruent conditions to verify that participants did not have difficulty in identifying any of the syllables. This setup also prevents participants from expecting any difference in the auditory-only or visual-only conditions^8^, as the proportion of the congruent trials were the same as the incongruent trials, unlike the biased proportion (8 congruent, 56 incongruent) used before (e.g.^24^).

We also avoided any possible suggestions to focus on either the visual or auditory domain when instructing participants. We asked participants to report “what did the person say?” Yet, in the Proverbio et al.’s study^23^, participants were instructed to report “what they had heard”. This can potentially lead to assigning more weight to the auditory cue than the visual one^2,41^. And in the Politzer-Ahles and Pan’s study^24^, participants were asked to report “what sound they *believed* they heard” The suggestive tone of “believe” may lead to expectation or bias to either modality.

Music training is known to narrow down the audiovisual integration time window^14^ and give an advantage in learning new auditory but not visual tasks^42^. Nevertheless, this was only observed in the music task but not in the speech task^17^. Indeed, despite the seemingly close relation between speech and music perception^43^, they rely on different acoustic cues and have their respective specializations in the auditory cortex^44^ and in other brain regions^45^. Neuroimaging studies have also shown that speech perception is more left-lateralized whereas music perception is more right-lateralized^46–48^. This may explain why musicians are more sensitive to audiovisual integration in the temporal domain. In the current study, however, there was no such difference in the susceptibility of the McGurk effect. This suggests that the McGurk effect is a speech-related perception phenomenon which may not overlap with music perception mechanisms^3^.

The conclusions reached in this study might be limited by the fact that our 73 participants may not cover the whole music training spectrum, specially at the extremes (e.g., people with absolute pitch). Future studies could explore this possibility.

In conclusion, the current study assessed different aspects of the music learning experience through the Gold-MSI and showed that musical expertise did not predict the susceptibility to the McGurk effect. We proposed several reasons why we had inconsistent findings with the previous two studies (which show contradictory results) and highlighted the importance to consider the different facets of the music learning experience, rather than focusing only on formal music training when evaluating its relation with perceptual processing. The difference between music perception and speech perception should be considered when conducting audiovisual integration studies and exploring how individual differences contribute to performance.

### Supplementary Information

Supplementary Table S1.

## Data Availability

Raw data is available on https://osf.io/nrdf6/.
