# Supplementary material for: Audiovisual integration in the McGurk effect is impervious to music training
Source: Sci Rep. 2024 Feb 8;14:3262. doi: 10.1038/s41598-024-53593-0 (PMC10853564; doi:10.1038/s41598-024-53593-0)
Supplement: Supplementary file 1 — Supplementary Table S1. [file 41598_2024_53593_MOESM1_ESM.pdf]

Supplementary

Table S1. The mean scores of Gold-MSI in the previous and current studies

	Active engagement	Perceptual abilities	Music training	Singing skills	Emotions	General sophistication
Norm; Müllensiefen et al. (2014)	42	50	27	32	35	82
Correia et al. (2022)	39.24	49.77	27.72	30.94	34.5	79.38
Lima et al. (2020)	33.03	44.55	18.48	26.67	31.32	64.8
Rimmele et al. (2022)	35.78	47.34	22.45	28.34	33.56	71.34
Current study	39.6	44.97	24.63	28.49	31.96	74.64
